# Possible Overestimation of Seed Transmission in the Spread of Pospiviroids in Commercial Pepper and Tomato Crops Based on Large-Scale Grow-Out Trials and Systematic Literature Review

**DOI:** 10.3390/plants10081707

**Published:** 2021-08-19

**Authors:** Jacobus T. J. Verhoeven, Marleen Botermans, Ruben Schoen, Harrie Koenraadt, Johanna W. Roenhorst

**Affiliations:** 1 National Plant Protection Organization of the Netherlands, P.O. Box 9102, 6700 HC Wageningen, The Netherlands; koverhoeven@gmail.com (J.T.J.V.); m.botermans@nvwa.nl (M.B.); r.schoen@nvwa.nl (R.S.); 2 Naktuinbouw Research and Development, P.O. Box 40, 2370 AA Roelofarendsveen, The Netherlands; h.koenraadt@naktuinbouw.nl

**Keywords:** commercial seed lots, *Capsicum annuum*, epidemiology, source of infection, outbreak, pathway, plant disease management, *Solanum lycopersicum*

## Abstract

Several outbreaks of pospiviroids have been reported in pepper and tomato crops worldwide. Tracing back the origin of the infections has led to different sources. In some cases, the infections were considered to result from seed transmission. Other outbreaks were related to transmission from ornamental crops and weeds. Pospiviroids, in particular potato spindle tuber viroid, are regulated by many countries because they can be harmful to potatoes and tomatoes. Seed transmission has been considered an important pathway of introduction and spread. However, the importance of this pathway can be questioned. This paper presents data on seed transmission from large-scale grow-out trials of infested pepper and tomato seed lots produced under standard seed-industry conditions. In addition, it presents the results of a systematic review of published data on seed transmission and outbreaks in commercial pepper and tomato crops. Based on the results of the grow-out trials and review of the literature, it was concluded that the role of seed transmission in the spread of pospiviroids in practice is possibly overestimated.

## 1. Introduction

Over the last few decades, several outbreaks of pospiviroids in pepper (*Capsicum annuum*) and tomato (*Solanum lycopersicum*) have been reported worldwide (reviewed by Candresse et al. [1] and Hammond [2]). Pospiviroids, and in particular potato spindle tuber viroid (PSTVd), are regulated in many countries because of their harmful effects on potato (*Solanum tuberosum*) and tomato crops [2,3,4]. Phytosanitary measures are taken to prevent and eradicate outbreaks, and efforts are made to trace the sources of infection. With regard to the outbreaks in pepper and tomato crops, both the introduction of pospiviroids, via infested seed lots, and transfer from infected ornamental crops and weeds, have been reported [2]. However, the relative importance of each of these sources as a pathway for introduction in pepper and tomato crops worldwide is unclear.

Pospiviroids are single-stranded circular RNA molecules of ca. 360 nucleotides able to infect plants. The genus *Pospiviroid*, one of the five genera within the family *Pospiviroidae*, includes nine species, of which *Potato spindle tuber viroid* is the type member [5,6]. All pospiviroid species, except iresine viroid 1, are able to infect at least one of the main solanaceous crops, i.e., pepper, potato, and tomato. In addition, pospiviroid infections have been reported from several ornamental crops and weeds, including many solanaceous species. However, pospiviroid infections in ornamentals often remain symptomless, which implies that these plants may serve as unnoticed sources of infection [7].

Pospiviroids can be spread by vegetative propagation and transmission via mechanical transfer (contact), insects, pollen, and seeds [1,4]. The role of vegetative propagation for spreading in potato and ornamental crops such as *Solanum jasminoides* is evident. Under experimental conditions, pospiviroids have been shown to be easily transferred mechanically [8,9]. In addition, in practice, the occurrence of spread by mechanical transfer is clear. Infections in tomato crops were found to spread rapidly within a row [10,11,12] and evidence was obtained that outbreaks in pepper and tomato crops were the result of mechanical transfer from symptomless-infected ornamentals and weeds, e.g., [13,14]. Regarding the role of insects in the spread of pospiviroids, aphids have been found to transmit PSTVd in mixed infections with potato leaf roll virus (PLRV) in potatoes [15,16], and tomato chlorotic dwarf viroid (TCDVd) with PLRV in tomatoes [17]. In addition, pollination by bumblebees has been associated with the spread of tomato apical stunt viroid (TASVd) [18,19] but was not observed for PSTVd [20]. The latter results [20] also indicated that the role of pollen in the spreading of pospiviroids is limited. Finally, seeds have been reported as a source of infection for seed-propagated crops such as pepper and tomato. However, reports on seed transmission in these crops are contradictory. Under experimental conditions, substantial transmission rates, as well as the complete absence of seed transmission, have been reported, e.g., [21,22]. For outbreaks in commercial crops, some were considered to result from infested seeds [23,24], whereas for others, no evidence was found for seed as a source of infection [12]. The rationale behind these different observations is not understood, and possible explanations include viroid load, ‘age’ and/or treatment of seeds, and climatic conditions (temperature) during germination and cultivation.

Several countries consider seeds as an important pathway for the introduction of pospiviroids in commercial pepper and tomato crops. Because production and processing occur at a global scale, this has led to installing strenuous seed-testing requirements for seed lots to be traded. However, the importance of seeds as a pathway for the introduction of pospiviroids in commercial crops has never been evaluated. Which part of the outbreaks can be attributed to the introduction via seed versus mechanical transfer from (symptomless) infected ornamentals and weeds, and what is the evidence?

To determine the role of seeds as a pathway for the introduction of pospiviroid infections in pepper and tomato crops, grow-out trials under standard conditions were performed using seed lots naturally infested by columnea latent viroid (CLVd), pepper chat fruit viroid (PCFVd), and PSTVd. In addition, published data were reviewed on the evidence of seed transmission under experimental conditions and in practice. The results of the grow-out trials, as well as the reviews of published data, will be discussed in view of the role of seeds as a pathway for the introduction of pospiviroids in commercial pepper and tomato crops.

## 2. Materials and Methods

### 2.1. Grow-Out Trials

#### 2.1.1. Selection of Seed Lots

For the grow-out trials, commercially produced seed lots were selected that tested positive for pospiviroids during routine screening by real-time RT-PCR according to the EPPO standard PM7/138 [25], Appendix 4. To identify the seed lots with the highest viroid loads for each of these lots, three subsamples of 1000 seeds and eight subsamples of 100, 10, and 1 seed(s) were tested. The data was used to estimate the infestation rate using the software package SeedCalc8 (version 8.1.0; Geves, France). The lots with the highest viroid loads and the most consistent test results were selected for the grow-out trials. The selected seed lots concerned seeds from different cultivars and seed companies. They had been produced and initially processed in either Africa or Asia and were further processed in Europe, according to standard procedures. Except for a 1%-HCl treatment during the extraction of the tomato seeds, which is compulsory in the European Union, no additional treatments were applied for disinfection.

#### 2.1.2. Confirmation of the Identity and Viability of the Viroids in the Selected Seed Lots

To confirm the presence and identity of the viroids detected during routine screening, conventional RT-PCRs were performed on RNA extracts using the following primers: pCLV4/pCLVR4 for CLVd [26]; Pospi1FW/RE and AP-FW1/RE2 for PCFVd [12,27], Pospi1FW/RE and Pospi2FW/RE for PSTVd lot 3, and Pospi1FW/RE for PSTVd lot 5 [12,28]. Amplicons were bi-directionally sequenced and analysed [29].

To determine the viability of the viroids, for each seed lot RNA extracts were mechanically inoculated onto four plants of *C. annuum* cv. Westlandse Grote Zoete and four plants of *S. lycopersicum* cv. Moneymaker according to Verhoeven et al. [28]. After six weeks, the inoculated pepper and tomato plants were tested in groups of four by real-time RT-PCR [30].

#### 2.1.3. Conditions Grow-Out Trials

The grow-out trials of both the infested pepper and tomato seed lots were performed at seven different locations in the Netherlands. Three seed lots were grown in dedicated greenhouses of six seed companies and two seed lots in the quarantine greenhouse of the National Plant Protection Organization (NPPO) of the Netherlands. To prevent viroid transmission to and from external hosts, all treatments were performed under containment conditions.

Between 12 and 17 days after germination, when the first fully developed leaf had appeared in most seedlings, plants were manually transplanted in rock-wool blocks or potting soil. Plants were grown for 56–59 days at a minimum temperature of 25 °C and a day length of at least 14 h. Common cultivation practices such as irrigation and insect control were applied, but pruning was limited and aberrant plants were not removed.

#### 2.1.4. Detection of Pospiviroids in Leaf Samples from Seedlings

At the end of the grow-out trials, the top leaflet of a young (50–90% full-grown) leaf of each seedling was collected. Equal parts of leaves were pooled up to 25 and tested for pospiviroids at the laboratories of the participating seed companies or Naktuinbouw, using routine procedures adapted from Botermans et al. [30].

### 2.2. Review of Published Data on Seed Transmission and Outbreaks

Data on seed transmission and outbreaks of pospiviroids in pepper and tomato crops were extracted from publications in scientific journals and the EPPO Reporting Service, the latter only when data on outbreaks were not published elsewhere. In addition, data of the grow-out trials reported in this paper was included for seed transmission. For reviewing, publications were grouped per crop and considered per viroid species. Regarding seed transmission, the following data were considered: type of seed (produced experimentally or commercially), number of infected/raised seedlings, and specific conditions if applicable. For reviewing the data on outbreaks, the term outbreak had been defined as the report of a single viroid per crop and country. For each outbreak, the evidence for the indicated source of infection was considered in relation to the stage of the crop at the time of viroid detection, the genome sequence of the isolate, information on other crops grown from the same seed lot, environmental conditions, etc. Based on an ‘unbiased’ review of these data, conclusions were drawn on the most probable source(s) of infection. These conclusions were compared with those given by the authors.

## 3. Results

### 3.1. Grow-Out Trials

#### 3.1.1. Confirmation of the Identity and Viability of the Viroids in the Selected Seed Lots

For the selected seed lots, the presence and identity of the pospiviroids were confirmed by sequencing and analysis of the amplicons produced by RT-PCR. Table 1 shows the identified viroids and accession numbers in NCBI GenBank. For seed lots 1 to 4, complete genome sequences of the viroid isolates were obtained, except for the primer positions. For lot 5, only a partial sequence of 112 nt of PSTVd was obtained.

For the viroid isolates from the three pepper seed lots, the viability was shown by successful infection of *C. annuum* cv. Westlandse Grote Zoete and *S. lycopersicum* cv. Moneymaker after mechanical inoculation (results not shown). For the viroid isolates from the two tomato seed lots, the viability could not be confirmed. These results show that at least for pepper, the viability of the viroids was maintained during the processing and storage of the seeds, whereas for tomatoes such a conclusion could not be drawn.

#### 3.1.2. Detection of Pospiviroids in Leaf Samples from Seedlings

Table 2 shows the number of pepper and tomato seedlings included in the grow-out trials and their distribution over the seven locations. Up to 56–59 days after sowing, no viroid symptoms were observed and testing of all plants at the end of the growing period did not reveal any viroid infection. This means that in both crops, CLVd, PCFVd, and PSTVd were not transmitted from seeds to seedlings/plants even though the environmental conditions were favourable for viroid transmission and replication.

### 3.2. Review of Published Data on Seed Transmission and Data from This Study

A total of 27 publications on seed transmission of pospiviroids were considered and reviewed per crop and type of seed production. The results are summarised in Table 3, Table 4, Table 5 and Table 6 and further detailed in Supplementary Materials Table S1 (A–D).

#### 3.2.1. Pepper

For experimentally produced pepper seeds, seed transmission has been reported for PCFVd and PSTVd (Table 3). Verhoeven et al. [27] reported transmission of PCFVd for 11 out of 59 seedlings. However, Verhoeven et al. [31] were not able to confirm the previously reported seed transmission for PCFVd in 179 seedlings of the same pepper variety and 158 seedlings of another variety. In addition, no seed transmission of PCFVd was reported in 46 seedlings by Yanagisawa and Matsushita [32]. For PSTVd, Matsushita and Tsuda [33] reported transmission for seven out of 2230 seedlings. In contrast, no seed transmission was reported by Lebas et al. [34] and by Verhoeven et al. [31] for 25 and 222 pepper seedlings. With regard to the other pospiviroids, no seed transmission was reported for columnea latent viroid (CLVd) [31], TASVd [31], TCDVd [33], and tomato planta macho viroid (TPMVd) [32], based on examination of 179, 217, 1105, and 46 seedlings, respectively. For commercially produced pepper seeds, the grow-out trials reported in this paper did not show seed transmission for CLVd, PCFVd, and PSTVd in 27,703, 55,438, and 2500 seedlings, respectively. Furthermore, Verhoeven et al. [28] did not observe seed transmission for TASVd in 1200 seedlings (Table 4).

**Table 3:** The number of infected/raised pepper seedlings in ten separate grow-out trials of experimentally produced seeds reported in the literature.

CLVd		PCFVd		PSTVd		TASVd		TCDVd		TPMVd	
0	179 ^a^	11	59 ^b^	0	25 ^d^	0	217 ^a^	0	1105 ^e^	0	46 ^c^
		0	337 ^1a^	7	2230 ^2e^						
		0	46 ^c^	0	222 ^a^						

^1^ Total of two varieties; ^2^ total of three varieties; ^a^ [31]; ^b^ [27]; ^c^ [32]; ^d^ [34]; ^e^ [33].

**Table 4:** The number of infected/raised pepper seedlings in four separate grow-out trials of commercially produced seeds reported in the literature and in this paper.

CLVd		PCFVd		PSTVd		TASVd	
0	27,703 ^a^	0	55,438 ^1a^	0	2500 ^a^	0	1200 ^b^

^1^ Total of two varieties; ^a^ this study; ^b^ [28].

#### 3.2.2. Tomato

Regarding experimentally produced tomato seeds, seed transmission has been reported for all seven pospiviroids found in commercial tomato crops (Table 5). However, for most pospiviroids results were contradictory. For citrus exocortis viroid (CEVd), Semancik [35] reported seed transmission in tomato without further details, whereas Faggioli et al. [21] did not find transmission for 1849 seedlings. For CLVd, Matsushita and Tsuda [33] reported transmission for 46 out of 793 seedlings, whereas no seed transmission was found by Fox and Monger [36] and Faggioli et al. [21] for 200 and 1599 seedlings. For PCFVd, Yanagisawa and Matsushita [32] reported three infected seedlings out of 941. For PSTVd, again, contradictory results have been reported. Successful seed transmission was reported by Khoury et al. [37], Singh and Dilworth [38], Simmons et al. [22] and Matsushita and Tsuda [33] for 459 out of 1933 seedlings in total. In addition, seed transmission without further details was reported by Benson and Singh [39], Kryczynski et al. [40], Menzel and Winter [41], and Batuman et al. [42]. In contrast, Lebas et al. [34] and Faggioli et al. [21] did not find seed transmission of PSTVd for a total of 47 seedlings, neither did McClean [43] for tomato bunchy top virus (synonym PSTVd) in 92 seedlings. For TASVd seed transmission was reported for 24 out of 30 seedlings by Antignus et al. [18] and without further details by Batuman et al. [42], but no transmission was found by Faggioli et al. [21] and Matsushita and Tsuda [33] for 2575 seedlings. For TCDVd, Singh and Dilworth [38] reported seed transmission for 209 out of 280 seedlings, whereas no transmission was found in over 4251 seedlings by Singh et al. [44], Koenraadt et al. [45] and Matsushita and Tsuda [33]. Finally, for TPMVd, seed transmission was reported by Yanagisawa and Matsushita [32] for 13 out of 1039 seedlings, but not by Belalcazar and Galindo-Alonso [46] for 425 seedlings. Overall, 724 seedlings were found infected among a total of 16,054 raised plants, the efficiency of the seed transmission varying from 0 to 80%.

**Table 5:** The number of infected/raised tomato seedlings in 22 separate grow-out trials of experimentally produced seeds reported in the literature.

CLVd		CEVd		PCFVd		PSTVd		TASVd		TCDVd		TPMVd	
0	1599 ^1,a^	0	1849 ^1,a^	3	941 ^d^	0	22 ^a^	24	30 ^k^	0	4000 ^l^	0	425 ^n^
0	200 ^b^					3	60 ^e^	0	1232 ^1,a^	0	251^c^	13	1039 ^d^
46	793 ^2c^					0	25 ^f^	0	1343 ^3,c^	0	? ^m^		
						111	285 ^4,c^			209	280 ^j^		
						0	92 ^5,g^						
						178	350 ^h^						
						107	1192 ^i^						
						30	46 ^j^						

^1^ Total of two varieties; ^2^ total of four varieties; ^3^ total of three varieties; ^4^ total of five varieties; ^5^ results for tomato bunchy top virus (synonym PSTVd [43]); ^a^ [21]; ^b^ [36]; ^c^ [33]; ^d^ [32]; ^e^ [37]; ^f^ [34]; ^g^ [43]; ^h^ [22]; ^I^ [47]; ^j^ [38]; ^k^ [18]; ^l^ [45]; ^m^ [44]; ^n^ [46]. Seed transmission without further details was reported by Benson and Singh [39] (PSTVd), Semancik [35] (CEVd), Kryczynski et al. [40] (PSTVd), Menzel and Winter [41] (PSTVd), and Batuman et al. [42] (PSTVd and TASVd).

For commercially produced tomato seeds, also successful and unsuccessful seed transmission have been reported (Table 6). In comparison to experimentally produced seeds, both frequency and the rate of seed transmission were low, i.e., one out of 370 seedlings for PSTVd by Van Brunschot et al. [24] and 2–20 out of 2500 seedlings for TCDVd by Candresse et al. [23]. In the latter case, the viroid was detected in two out of 250 samples by testing bulked samples of 10 seedlings, explaining the range of 2–20 seedlings. In the grow-out trials reported in this paper, no seed transmission of PSTVd was found for 2500 seedlings. Similarly, no transmission was found for 1000 seedlings in a previous trial, where the seed was harvested from an infected crop according to commercial practices [48]. For CLVd and PCFVd, no seed transmission was found for 25,500 [36] and 47,477 tomato seedlings (this study). Overall, for a total of 79,398 tomato seedlings only in two cases was seed transmission reported.

**Table 6:** The number of infected/raised tomato seedlings in six separate grow-out trials of commercially produced seeds reported in the literature and in this paper.

CLVd		PCFVd		PSTVd		TCDVd	
0	25,500 ^a^	0	47,528 ^b^	1	370 ^c^	2–20 ^1^	2500 ^e^
				0	1000 ^d^		
				0	2500 ^b^		

^1^ Two out of 250 bulked samples of 10 seedlings tested positive; ^a^ [36]; ^b^ this study; ^c^ [24]; ^d^ [48]; ^e^ [23].

### 3.3. Review of Published Data on Outbreaks

#### 3.3.1. Pepper

For pepper, six publications on at least 14 outbreaks of pospiviroids were reviewed with regard to the source of infection (Table 7). In five cases, the current review resulted in the same conclusion as drawn by the authors, i.e., the most probable origin being other host plants or unknown (Table 7; Supplementary Materials Table S2A). Only in one case in New Zealand, where the authors assumed seeds as the source of the PSTVd infection [34], the conclusion could be questioned. Firstly, none of the 25 seedlings grown from seeds of the infected pepper plants was found to be infected. Secondly, highly similar PSTVd sequences were found in four pepper varieties in five different glasshouses, as well as in tomato and cape gooseberry crops grown in this country [34,49,50]. Together, these observations are reasons to consider locally infected host plants as a more probable source of infection. For similar reasons, Mackie et al. [51] considered local wild plants as the source of infection of repeated PSTVd outbreaks in pepper crops in Western Australia.

#### 3.3.2. Tomato

For tomato, 43 publications on outbreaks of pospiviroids were reviewed (Table 8; Supplementary Materials Table S2B). Thirty-nine publications concerned one viroid, three reported on two viroids [42,55,56], and one publication reported on four pospiviroids, of which one was reported from two countries without obvious connection to each other [12]. This makes a total of 50 outbreaks. For 11 out of these 50 pospiviroid outbreaks in tomatoes, the authors indicated seeds as the most likely source of infection. Upon review, in nine cases the provided data were insufficient to substantiate this conclusion [18,49,57,58,59,60], [42] (two outbreaks), [55] (one outbreak), and seeds seemed to be indicated as a source of infection by default. For seven outbreaks too little information was provided to conclude on the source of infection and, therefore, upon review it was considered unknown. In two cases plants were considered as the most probable source of infection, instead of seeds. Firstly, in 2002 Antignus et al. [61] reported on the spread of TASVd from a few infected plants in a tomato crop and indicated that the origin of the infections in these plants was unknown. In 2007, the same authors reporting on the same outbreak suggested seeds as the most probable source of infection [18]. The latter conclusion was based on the demonstration of seed transmission under experimental conditions for seed batches not related to the outbreak. Secondly, Elliott et al. [49] indicated seeds as the most probable source of the PSTVd outbreak in a tomato crop in New Zealand in 2000, without providing data to support this suggestion. Since the first symptomatic plants were found near the entrance of the glasshouse, and the viroid sequence was almost similar to that of other PSTVd isolates reported in tomato, pepper and *Physalis peruviana* in New Zealand [34,50], infected host plants in the environment seem a more probable source of infection. In only two publications, data were provided indicating that seeds could have been the source of infection of the PSTVd and TCDVd infections in tomatoes [23,24]. For ten outbreaks, infected plants were considered as the most probable source of infection [12] (three outbreaks) [13,51,62,63,64,65,66]. Reviewing the data lead to the same conclusion in nine cases. For the outbreak in Germany [63], however, it was concluded that the data provided did not allow to conclude on the source of infection. In the remaining 29 outbreaks in tomato (27 publications), no conclusion was drawn on the source of infection. For three of these publications, reviewing the data led to the conclusion that infected host plants were the most probable source of infection. Firstly, Ling and Sfetcu [67] reported the source of the PSTVd outbreak in tomatoes in California (USA) in 2009 to be unknown, although tomato plants in the affected glasshouse and its surroundings were already known to be infected by PSTVd for several years. Therefore, these infected plants were considered the most probable source of the infections in 2010 (Verhoeven, unpublished data). Secondly, for the outbreak reported by Mackie et al. [10], the genome sequence of the PSTVd isolate was found to show the highest identities with PSTVd sequences from the *P. peruviana* cluster, which also includes other isolates from wild plants in Australia [51,68]. The publication of Mackie et al. [51] provided further evidence that local plants most likely served as the source of infection. Thirdly, in Japan, infected petunia plants appeared to be the most probable source of infection of the TCDVd outbreak in tomatoes in 2008, since the genome sequence of the isolate identified in tomato (AB329668) [11] was reported from many petunia selections originating in Japan [69,70]. The latter conclusion was further supported by the observation that most TCDVd sequences from tomato crops outside Japan do not group in the cluster of petunia sequences [69]. For the remaining 26 outbreaks, no source of infection could be indicated, based on the data provided.

## 4. Discussion

The results of the grow-out trials of commercially produced pepper and tomato seeds, combined with the results of the reviews of data from publications on seed transmission and outbreaks, indicate that the role of seed transmission in the spread of pospiviroids in these crops may have been overestimated.

The results presented here show that none of the 57,938 pepper and 50,028 tomato seedlings raised from commercially produced infested seed lots was found infected, neither by symptom observation nor by testing. For pepper, the lack of seed transmission could not be ascribed to the absence of viable viroid, given the successful infection of healthy pepper and tomato plants after mechanical inoculation. For tomatoes, the viability of the viroids could not be established. The lack of successful infection of the inoculated plants does not seem to relate to the amount of viroid, since the estimated viroid load of one of the tomato seed lots was substantially higher than that of the pepper seed lots. An effect of the seed processing procedure on the viability of the viroid cannot be excluded, such as the HCl treatment, neither an effect of the matrix on the success of mechanical transmission. A matrix effect has been observed, e.g., for the transmission of PSTVd from leaf material of *Brugmansia* sp. in comparison to *Solanum jasminoides* [9,14,64]. In conclusion, these results indicate that for commercially produced seeds the chance is low that pospiviroids are transmitted from seed to seedling, even in the case that viable viroids are present as shown for pepper.

The lack of seed transmission in grow-out trials of commercially produced seed lots was further supported by a review of publications on seed transmission of pospiviroids in pepper (Table 4) and tomato (Table 6). Although the rate of seed transmission may be affected by the viroid variant, (a cultivar of) the host plant and environmental conditions, the substantially higher seed-transmission rates reported for experimentally produced seeds (Table 3 and Table 5) raised the question of whether the industrial seed processing also may affect the transmission from seed to seedling. For pepper seeds, Verhoeven et al. [31] did not find such an effect, when simulating industrial processing by postponing the date of sowing. Neither does industrial processing seem to abolish the viability of the viroids of infested seeds. For tomato seeds, the effect of seed processing on transmission cannot be excluded, since the viability of the viroid on infested tomato seeds could not be established. Nevertheless, the question remains why the figures of seed transmission of pospiviroids are so different for experimentally and commercially produced seeds. Could cross-contamination have occurred under experimental conditions where research with these highly contagious viroids is performed by staff working on various aspects of these pathogens while using the same facilities? For example, Verhoeven et al. [31] could not confirm the earlier seed transmission of PCFVd in pepper [27], despite using the same isolate and cultivar under the same growing conditions. Therefore, they questioned whether these different results could be attributed to the occurrence of cross-contamination in the previous experiments. More generally, it raises the question of whether cross-contamination could also have accounted for the establishment of infections in seedlings in other experiments? In relation to the TCDVd outbreak reported by Candresse et al. [23], in 4000 seedlings grown from seeds harvested from the affected crops, no infections were found by Koenraadt et al. [45]. In addition, for the single positive seedling reported by van Brunschot et al. [24], the identified genome sequence of PSTVd was highly similar to the majority of PSTVd genotypes reported in the region [10,51,68] and, therefore, does not exclude local plants as a source of infection. Nevertheless, the results of Candresse et al. [23] and Van Brunschot et al. [24], indicate that exceptional seed transmission of pospiviroids in tomatoes cannot be excluded.

Overall, the review of published data on pospiviroid outbreaks in pepper and tomato crops indicate that designating seed as a source of infection is often by default rather than evidence-based. Reviewing the publications in which seeds were suggested as a source of infection resulted in different conclusions in the only case for pepper and nine from eleven cases for tomatoes (Table 7 and Table 8). Regarding the two remaining publications suggesting seeds as the source of infection in tomato crops, at least some questions can be raised from this conclusion, as discussed before. In the majority of the publications, the source of infection was not specified or unknown, which did not change after review.

Finally, the conclusion that the role of seed transmission in the global spread of pospiviroids may be overestimated, is supported by the fact that the number of outbreaks in tomato and pepper crops has been limited even before the testing of seed lots was implemented. Constable et al. [89] reported the detection of pospiviroids in 36 out of 553 (6.5%) imported pepper seed lots and in 91 out of 1562 (5.8%) tomato seed lots in Australia since 2008. These seed lots were reported to originate from Africa, Asia (eastern and southern regions), Europe, the Middle East, as well as North and South America. In addition, in the Netherlands, pospiviroids were detected in 70 out of 2997 (2.3%) pepper and 140 out of 5874 (2.4%) tomato seed lots from various origins between 2015 and 2018 (Koenraadt, unpublished data). The recent detection of TASVd in a pepper seed lot produced in 1992, however, indicated that pospiviroids already occurred in these crops before testing started [28]. Therefore, far more reports of outbreaks would have been expected when seeds are a substantial source of infection. Nevertheless, in Australia, the number of reported pospiviroid outbreaks was limited, also before testing of imported seed lots started in 2008. For none of the reported cases from this period [10,59,68] were seeds considered the most probable source of infection in the current review (Table S2). For the two outbreaks in Australia since then, Mackie et al. [51] reported plants and Van Brunschot et al. [24] reported seeds as the most likely sources of infection. Moreover, in other countries, the number of reported pospiviroid outbreaks in tomato and pepper crops was low, and outbreaks were only occasionally assumed to be related to infested seed lots (Table 7 and Table 8, Table S2). Taken together, both the substantial number of infested seed lots found by Constable et al. [89] and Koenraadt (unpublished data) and the low number of pospiviroid outbreaks reported, support the conclusion that the role of seed transmission in the spread of pospiviroids in pepper and tomato crops has been overestimated.

## 5. Conclusions

In conclusion, this systematic review of published data on seed transmission and outbreaks in pepper and tomato, including the results of the grow-out trials described in this paper, sheds new light on the contradictory views on the contribution of seeds to the spread of posiviroids. These new insights can be used to reassess the role of seeds as a pathway for the spread of pospiviroids in these crops, and to develop and substantiate alternative disease management strategies at lower costs by avoiding unnecessary destruction of ‘infested’ seed lots.

## Figures and Tables

**Table 1:** Seed lots used for grow-out trials to determine the transmission from seeds to seedlings.

Lot Number	Crop ^1^	Origin	Viroid(s) Identified	Genbank Accession Number	Estimated Infestation Rate ^2^	Number of Plants Raised
1	pepper	Asia	PCFVd	MW422288	13% (CL_95_ 0–52%)	27,735
2	pepper	Asia	CLVd	MW422289	9% (CL_95_ 3–22%)	27,703
			PCFVd	MW422290	13% (CL_95_ 4–29%)	
3	pepper	Africa	PSTVd	MW422291	nd	2500
4	tomato	Asia	PCFVd	MW422292	63% (CL_95_ 25–91%)	47,528
5	tomato	Asia	PSTVd	MW422293	nd	2500

^1^ Seed lots concerned distinct pepper and tomato cultivars from different seed companies. ^2^ Based on SeedCalc8 (version 8.1.0; Geves, France); CL_95_: confidence level 95%; nd: not determined.

**Table 2:** The number of pepper and tomato plants raised from infested seed lots and distributed over the different locations.

Location ^1^	Pepper			Tomato	
	Lot 1	Lot 2	Lot 3	Lot 4	Lot 5
	PCFVd	CLVd and PCFVd	PSTVd	PCFVd	PSTVd
1	5575	5500		8256	
2	5805	6045		9985	
3	6000	6000		10,000	
4	5605	5358			
5	4750	4800		10,215	
6				9072	
7			2500		2500
Total	27,735	27,703	2500	47,528	2500

^1^ Locations 1–6: dedicated greenhouses at seed companies; location 7: quarantine greenhouse at the NPPO in the Netherlands.

**Table 7:** Conclusions on the most probable source of pospiviroid infections in pepper crops as reported and drawn upon in the current review of the provided data.

Source of Infection	Reported in Publication	Based on Review
Seeds	1 ^a^	0
Plants ^1^	4 ^b^	5
Unknown	1 ^c^	1

^1^ Plants include: plants for planting of the same crop and other hosts; ^a^ [34]; ^b^ [27,51,52,53]; ^c^ [54].

**Table 8:** Conclusions on the most probable source of pospiviroid infections in tomato crops as reported and drawn upon in the current review of the provided data.

Source of Infection	Reported in Publication	Based on Review
Seeds	11 ^a^	2
Plants ^1^	10 ^b^	14
Unknown	29 ^c^	34

^1^ Plants include: plants for planting of the same crop and other hosts. ^a^ Single outbreaks [18,23,24,49,55,57,58,59,60], two outbreaks [42]; ^b^ Single outbreaks [13,51,62,63,64,65,66], three outbreaks [12]; ^c^ Single outbreaks [10,11,44,46,55,61,67,71,72,73,74,75,76,77,78,79,80,81,82,83,84,85,86,87,88], two outbreaks [12,56].

## Data Availability

Data is contained within the article and the Supplementary Materials.

## Supplementary Materials

The following are available online at https://www.mdpi.com/article/10.3390/plants10081707/s1, Table S1: Pospiviroid seed transmission experiments in pepper and tomato, Table S2: Pospiviroid outbreaks in pepper and tomato crops.
