# Supplementary material for: Possible Overestimation of Seed Transmission in the Spread of Pospiviroids in Commercial Pepper and Tomato Crops Based on Large-Scale Grow-Out Trials and Systematic Literature Review
Source: Plants (Basel). 2021 Aug 19;10(8):1707. doi: 10.3390/plants10081707 (PMC8400851; doi:10.3390/plants10081707)
Supplement: Supplementary file 1 [file plants-10-01707-s001.zip › plants-1328463-supplementary.pdf]

Table S1.A. Seed transmission experiments using seeds from non-commercial seed lots of pepper

Reference	Citation	Viroid	Plants infected/ plants raised	Conditions	Comments
Lebas et al.,2005. Australasian Plant Pathology 34, 129-133.	34	PSTVd	0/25	25 C	Seedlings tested up to 27 weeks after germination.
Matsushita & Tsuda, 2016. EJPP 145, 1007-1011.	33	PSTVd	7/2,230	23-25 C	Total of three varieties.
Matsushita & Tsuda, 2016. EJPP 145, 1007-1011.	33	TCDVd	0/1,105	23-25 C	
Verhoeven et al., 2009. Virus Res 144, 209-214.	27	PCFVd	11/59	20-25 C	Viroid sequence had not changed after seed transmission.
Verhoeven et al., 2020. Eur J Plant Pathol 156, 21-29.	31	CLVd	0/168	25 C, at least 14h illumination	
Verhoeven et al., 2020. Eur J Plant Pathol 156, 21-29.	31	PCFVd	0/337	25 C, at least 14h illumination	Total of two varieties.
Verhoeven et al., 2020. Eur J Plant Pathol 156, 21-29.	31	PSTVd	0/222	25 C, at least 14h illumination	
Verhoeven et al., 2020. Eur J Plant Pathol 156, 21-29.	31	TASVd	0/217	25 C, at least 14h illumination	
Yanagisawa & Matsushita, 2017. EJPP 149, 211–217.	32	PCFVd	0/46	25-27 C, 16h illumination	Harvested seeds were directly sown.
Yanagisawa & Matsushita, 2017. EJPP 149, 211–217.	32	TPMVd	0/46	25-27 C, 16h illumination	Harvested seeds were directly sown.

Table S1.B. Seed transmission experiments using seeds from commercial seed lots of pepper

**Reference**	**Citation**	**Viroid**	**Plants infected/ plants raised**	**Conditions**	**Comments**
Verhoeven et al., 2017. Arch Virol 162, 1741-1744.	28	TASVd	0/1200	25 C, at least 14h illumination	Seeds originating from Taiwan.
Verhoeven et al., this publication.		CLVd	0/27,703	25 C, at least 14h illumination	Seeds originating from Asia.
Verhoeven et al., this publication.		PCFVd	0/55,438	25 C, at least 14h illumination	Total of two varieties. Seeds originating from Asia
Verhoeven et al., this publication.		PSTVd	0/2500	25 C, at least 14h illumination	Seeds originating from Africa.

Table S1.C. Seed transmission experiments using seeds from non-commercial seed lots of tomato

Reference	Citation	Viroid	Plants infected/ plants raised	Conditions	Comments
Antignus et al., 2007. Plant Disease 91, 47-50.	18	TASVd	24/30	22-35 C	Seed treatment by 10% TSP or 2% sodium hypochlorite prevented detection of TASVd but not transmission.
Batuman et al, 2019. Plant Disease 103, 1525-1535.	42	PSTVd	?/150	Not reported	The authors report that a small number of seedlings (<1%), germinated from seeds collected from PSTVd infected plants, had necrotic lesions in hypocotyls and cotyledonary leaves. These tissues tested positive for PSTVd. Furthermore, the authors also report < 1% infections by PSTVd in 10 samples (!) of randomly collected hypocotyls and cotyledonary leaves without symptoms. NB How can the infection rate of 10 samples be <1%? Detailed data, however, have not been reported. Moreover, in a internet slide presentation on these viroid outbreaks in Ghana, the senior author reports PSTVd and TASVd positive results in seed tests, but no data were presented on testing of seedlings (https://ipmil.cired.vt.edu/wp-content/uploads/2014/06/Tomato-infecting-viroids-West-African-Example.pdf. Data retrieved 20210319.) Finally, the authors report in the summary of the publication that both viroids were possibly seed transmitted! Therefore, it should be concluded that seed transmission was not conclusively demonstrated.
Batuman et al, 2019. Plant Disease 103, 1525-1535.	42	TASVd	?/150	Not reported	The authors report that a small number of seedlings (<1%), germinated from seeds collected from TASVd infected plants, had necrotic lesions in hypocotyls and cotyledonary leaves. These tissues tested positive for TASVd. Furthermore, the authors also report < 1% infections by TASVd in 10 samples (!) of randomly collected hypocotyls and cotyledonary leaves without symptoms. NB How can the infection rate of 10 samples be < 1%? Detailed data, however, have not been reported. Moreover, in a internet slide presentation on these viroid outbreaks in Ghana, the senior author reports PSTVd and TASVd positive results in seed tests, but no data were presented on testing of seedlings (https://ipmil.cired.vt.edu/wp-content/uploads/2014/06/Tomato-infecting-viroids-West-African-Example.pdf. Data retrieved 20210319.) Finally, the authors report in the summary of the

					publication that both viroids were possibly seed transmitted! Therefore, it should be concluded that seed transmission was not conclusively demonstrated.
Belalcazar & Galindo-Alonso, 1974. Agrociencia, vol. 18, pp. 79-88.	46	TPMVd	0/425	Not reported	350 seeds were sown immediately and 350 seeds were sown after storage for 45 days.
Benson & Singh, 1964. Am Pot J 41, 294.	39	PSTVd	7.9 - 11.1%	Not reported	Abstract, details are lacking. Numbers are lacking but 7.9% to 11.1% seed transmission was reported in tomato.
Faggioli et al., 2015. Eur J Plant Pathol 143, 613-617.	21	CEVd	0/1849	20-25 C, 12-14h illumination	Total results of two varieties.
Faggioli et al., 2015. Eur J Plant Pathol 143, 613-617.	21	CLVd	0/1599	20-25 C, 12-14h illumination	Total results of two varieties.
Faggioli et al., 2015. Eur J Plant Pathol 143, 613-617.	21	PSTVd	0/22	20-25 C, 12-14h illumination	
Faggioli et al., 2015. Eur J Plant Pathol 143, 613-617.	21	TASVd	0/1232	20-25 C, 12-14h illumination	Total results of two varieties.
Fox & Monger, 2011. Report PC294, HDC Kenilworth (UK), 24 pp.	36	CLVd	0/200	24 C, 16h	
Khoury et al., 1988. Phytopathology 78, 1331-1336.	37	PSTVd	3/60	Not reported	
Koenraadt et al., 2009. Phytopathology 99, 6 (Su.pplement) S66	45	TCDVd	0/4000	Not reported	Same viroid sequence as used by Candresse et al. 2010. Seeds were harvested (experimentally) from naturally infected plants in France.
Kryczynski et al., 1988. J Phytopathol 121, 51-57[1].	40	PSTVd	?	2 months: 25-30 C, 18h 1800 lux	Infected and non-infected seeds of two varieties had the same germination rate; no symptoms in seedlings after 2m; all samples of 10 plants (10?) from seedlings gave positive results in PAGE (cv. Rutgers) and 9 of 10 in cDNA hybridisation (cv. Najwzesniejszy).
Lebas et al.,2005. Australasian Plant Pathology 34, 129-133	34	PSTVd	0/25	25 C	Seedlings tested up to 27 weeks after germination.
Matsushita & Tsuda, 2016. Eur J Plant Pathol 145, 1007-1011.	33	CLVd	46/793	23-25 C	Total results of four varieties.
Matsushita & Tsuda, 2016. Eur J Plant Pathol 145, 1007-1011.	33	PSTVd	111/285	23-25 C	Total results of five varieties.
Matsushita & Tsuda, 2016. Eur J Plant Pathol 145, 1007-1011.	33	TASVd	0/1,343	23-25 C	Total results of three varieties.
Matsushita & Tsuda, 2016. Eur J Plant Pathol 145, 1007-1011.	33	TCDVd	0/251	23-25 C	

McClean, APD, 1948. Science Bulletin Dep Agric For. No 256, 28pp[2].	43	PSTVd?	0/92		NB Report is on tomato bunchy top virus, a synonym of PSTVd.
Menzel & Winter, 2010. Mitt Julius-Kuhn-Inst 428 392-393.	41	PSTVd	6-16%	Not reported	Brief summary; details are lacking.
Semancik, JS 1980, CEVd, Descriptions of Plant Viruses, 226.	35	CEVd	?		Transmitted (personal communication Semancik) but transmission rate was not reported.
Simmons et al., 2015. J Plant Pathol Microbiol 6, 275.	22	PSTVd	178/350	21 C, 15h illumination	Why were positive results for PSTVd only obtained in 62-69% of tested seeds? 100% would be expected. Seedlings tested positive already at the third true leaf stage. Seed had been treated with 10% HCl (in NL grow-out experiments 1%). Seeds had been planted after one night of drying.
Singh, 1970. Am Pot J 47, 225-227.	47	PSTVd	107/1192	Not reported	Results in the table (left) are from fresh seeds (5-8d). After storage of 12 and 48 weeks, 30 out of 1000 and 32 out of 132 seedlings were found infected. The low % of transmission after 12 w storage might have been caused by the fact that many plants were aberrant and were not tested! NB transmission rate was 9, 3 and 24% after using 1, 12 and 48 weeks old seeds. Viroid from 72 w old seeds could still be mechanically inoculated to tomato plants.<br>To test whether external contamination of seed with PSTVd would give rise to infected seedlings, two lots of healthy tomato seeds were immersed in expressed sap from PSTVd infected tomato plants, for 5 minutes. One lot was dried for 72 hours, while the other lot was planted immediately. In three trials, involving 324 seedlings, none of the plants became infected. In a similar trial using PSTVd infected potato sap, one of the 50 plants developed symptoms.
Singh et al., 1999. J Gen Virol 80, 2823-2828.	44	TCDVd	0/?	Not reported	Four hundred seedlings of 7 plant species had been tested, one of which was tomato. Seedlings were tested by r-PAGE (method detected PSTVd control from true potato seed).
Singh & Dilworth, 2009. Eur J Plant Pathol 123, 111-116.	38	TCDVd	209/280	Not reported	Authors explain the failure of transmission by Singh et al., 1999 because they had used a more sensitive detection method now (RT-PCR). NB r-PAGE used in 1999 was successful for PSTVd in/from true potato seed.
Singh & Dilworth, 2009. Eur J Plant Pathol 123, 111-116.	38	PSTVd	30/46	Not reported	

Yanagisawa & Matsushita, 2017. Eur J Plant Pathol 149, 211–217.	32	PCFVd	3/941	25-27 C, 16h illumination	Harvested seeds were directly sown. Total of four varieties: transmission was found in only one variety being the same in which transmission was found for TPMVd.
Yanagisawa & Matsushita, 2017. Eur J Plant Pathol 149, 211–217.	32	TPMVd	13/1039	25-27 C, 16h illumination	Harvested seeds were directly sown. Total of four varieties: transmission was found in only one variety being the same in which transmission was found for PCFVd.

[1]CSVd and HSVd have not been included in this review. CSVd does not easily infect tomato (Hollings & Stone, 1973. Ann Appl Biol 74, 333-348). HSVd is a hostuviroid, not a pospiviroid. Moreover, no natural infections of these viroids in pepper and tomato have been reported.

Table S1.D. Seed transmission experiments using seeds from commercial seed lots of tomato

Reference	Citation	Viroid	Plants infected/ plants raised	Conditions	Comments
Candresse et al., 2010. Plant Dis 94, 633.	23	TCDVd	2-20/2,500	Not reported	Two of 250 subsamples of 10 seedlings each had tested positive. Further subsampling was not done, but considering the low number of positive subsamples, a maximum of one seedling per subsample is assumed to be potentially infected. Viroid sequence identical to the sequence found in the tomato greenhouse in France. Origin of seeds not reported.
Fox & Monger, 2011. Report PC294, 24 pp.	36	CLVd	0/25,500	24 C, 16h	Origin of seeds not reported.
Van Brunschot et al., 2014. Eur J Plant Pathol 139, 1-7.	24	PSTVd	1/370	Not reported	The seedlings had been tested 10 weeks after sowing; one symptomatic plant (shortened internodes and leaf purpling) was found positive. The viroid sequence had not changed after seed transmission. Origin of seeds not reported.
Verhoeven et al., 2015. Poster IWGLVV Haarlem.	48	PSTVd	0/1,000	25 C, at least 14h illumination	Origin of seeds not reported.
Verhoeven et al., this publication.		PCFVd	0/47,528	25 C, at least 14h illumination	The seeds had been treated with 1% HCl for 30 min; total of two varieties. Seeds originating from Asia.
Verhoeven et al., this publication.		PSTVd	0/2,500	25 C, at least 14h illumination	Seeds originating from Asia.

Table S2.A. Outbreaks in pepper crops

Reference	Citation	Country	Viroid	Outbreak number	Assumed source of infection[1]			Deduced source of infection[2]			Comments
					Seed	Plant	Unknown	Seed	Plant	Unknown	
EPPO Reporting Service 2016/084.	52	Switzerland	PSTVd	1		P			P		Outbreak originates from the same source as reported by Verhoeven et al., 2016.
Lebas et al.,2005. Australasian Plant Pathology 34, 129-133.	34	New Zealand	PSTVd	2	S				P		Infections in five of 43 inspected glasshouses. Authors suggest seeds as origin of the infections, but they failed to show seed transmission. The same viroid sequence was isolated from four pepper varieties in five glasshouses and was also similar to the sequences from tomato isolated in New Zealand before (Elliott et al., 2001) and from cape gooseberry Physalis peruviana isolated afterwards (Ward et al., 2010). These data suggest local plants as source of infection.
Mackie et al., 2016. Eur J Plant Pathol 145, 433-446.	51	Australia	PSTVd	3		P			P		Slightly different sequences all belonging to the cluster of Physalis peruviana were found in various tomato and pepper crops, as well as in samples of nine species of weeds and wild plants such as Physalis angulata. According to the authors, the naturally occurring PSTVd inoculum reservoir in wild plants and weeds explains the occurrence of repeated PSTVd infections in different years in field crops of tomato, pepper and chili.
Verhoeven et al., 2009. Virus Research 144, 209-214.	27	The Netherlands	PCFVd	4		P			P		Allthough this viroid was reported to be transmitted via seed, seeds were not assumed to be the source of infection. 1) The infection was only observed near the end of the growing season, 2) the disease was only found in one crop of pepper cv Jaguar, whereas 40 hectares of this variety had been grown in The Netherlands without viroid symptoms and 3) other solanaceous

											plants became infected after mechanical inoculation, showing that infections by PCFVd are not restricted to pepper, which implies that other plants species may have source of inoculum.
Verhoeven et al., 2011. New Disease Reports 23, 15.	54	Canada	PCFVd	5			U			U	Outbreak was assumed not to be correlated with the PCFVd outbreak in variety Jaguar in The Netherlands or with seeds. The viroid infected crop in Canada was of another variety than the in the Netherlands infected variety Jaguar, which moreover was produced from seeds of another seed company.
Verhoeven et al., 2016. New Disease Reports 34, 12.	53	The Netherlands	PSTVd	6		P			P		PSTVd infections had been identified in vegetatively-propagated pepper crops at four locations. The infections were correlated to the way of propagation, the first infected cutting originating from Israel.
					2	4	1	0	5	1	

[1] Source of infection assumed by the authors. 'Seed' covers the seeds from an infested lot. 'Plants' covers both infected plants for planting of the infected crop and infected wild or cutivated plants, not belonging to the plants of the infected crop. 'Unclear' is used when no source of infection is reported or in case of contradictory data.

[2] Deduced source of infection based on new interpretation of past and current conceptions. Further details are given in the column 'Comments'.

Table S2.B. Outbreaks in tomato crops

Reference	Citation	Country	Viroid	Outbreak number	Assumed source of infection[1]			Deduced source of infection[2]			Comments
					Seed	Plant	Unknown	Seed	Plant	Unknown	
Antignus et al., 2002. Phytoparasitica 30, 502-510.	61	Israel	TASVd	1			U			U	The first outbreak of TASvd is considered here. Same outbreak as reported by Antignus et al. (2007). Authors suggest that the tomato plants became infected from some initial foci of unknown origin. They also write that seed transmission - reported for some other viroids but not TASVd - may also account to the spread of the viroid to different geographical areas. It should be noted that since 1999 yearly outbreaks were recorded in the coastal region of Israel, indicating that except for the first outbreak, infected plants (tomatoes, ornamentals or wild plants) could have been the source of infection. Regarding the first outbreak both seeds and plants could have been the source.
Antignus et al., 2007. Plant Disease 91, 47-50.	18	Israel	TASVd	2	S				P		Various outbreaks of the same group of TASVd genotypes as reported in Antignus et al. (2002). Additional experiments were done and the viroid was detected in seeds from inoculated tomato plants and was transmitted via these seeds to seedlings (80%). Now, seed was considered the primary source of infection for the outbreak in tomato, however, substantial evidence is lacking. In addition, local introduction and/or spread of the viroid via bumble bees is also being considered. However, authors report yearly outbreaks in Israel, indicating that infected plants have been the source infection. In contrast, in other countries no outbreaks were recorded till the outbreak in Tunesia in 2006 (Verhoeven et al., 2006), whereas such outbreaks would not likely have passed unnoticed, due to the severe symptoms in tomato the viroid usually evokes. Therefore, plants have

											been the most likely source of inoculum for the infected Israelian tomato crops with the exception of th first outbreak for which the origin of infection is unclear.
Batuman et al., 2019. Plant Disease 103, 1525-1535.	42	Ghana	PSTVd	3	S					U	Four isolates of PSTVd from Ghana were identical and most closely related to PSTVd isolates from Turkey, New Zealand and the U.K., indicating that the PSTVd genotype from Ghana shows highest identies with sequences belonging to the cluster of sequences from Physalis peruviana (Verhoeven et al., 2010b). Because of the identical sequence of four PSTVd isolates from Ghana and the demonstrated seed transmissability, the authors assumed that PSTVd was inroduced in Ghana via seed. However, for a single introduction all four infections should have been introduced in the same variety and same seeds, but data are lacking. In addition, considering that Physalis peruviana may have been introduced to Oceania via Africa (Verhoeven at al., 2004; Verhoeven et al., 2010b), implies that native plants may have been sources of infection for the PSTVd-outbreaks in Ghana. Moreover, since rasta symptoms caused by different pospiviroids were found at various locations in Ghana in 2012 , indicates that PSTVd might have become established in Ghana in 2012. NB In a slide show from june 2014, the senior author reports viroid detection in tomato seeds extracted from viroid infected fruits, but not in seedlings grown from these seeds.
Batuman et al., 2019. Plant Disease 103, 1525-1535.	42	Ghana	TASVd	4	S					U	All four TASVd sequences from tomato plants in Ghana were different, however, they were most closely related to two isolates from Senegal and one from the USA. The authors assumed that all TASVd isolates were introduced into Ghana via seeds, because of the genetic diversity and the demonstrated seed transmissibility. However,

											since similar TASVd genotypes have been reported West Africa the viroid may have been established there too. Therefore, the origin of infection is unclear. NB In a slide show from june 2014, the senior author reports viroid detection in tomato seeds extracted from viroid infected fruits, but not in seedlings grown from these seeds.
Batuman & Gilbertson, 2013. Plant Disease 97, 692-693.	57	Mali	CLVd	5	S					U	CLVd was detected in seeds from inoculated tomato plants; however, seedlings were not tested, nor seeds and seedlings from the 'original' seed lot.
Belalcazar & Galindo-Alonso, 1974. Agrociencia 18, 79-88.	46	Mexico	TPMVd	6			U			U	No data on origin of the disease. Not transmitted to 425 seedlings.
Candresse et al., 1987. Nucleic Acids Research 15, 10597.	72	Indonesia	TASVd	7			U			U	Short paper, only reporting on the nucleotide sequence.
Candresse et al., 2007. PLant Dis 91, 330.	73	Senegal	TASVd	8			U			U	No information on possible origin of the viroid.
Candresse et al., 2010. Plant Dis 94, 633.	23	France	TCDVd	9	S			S			Two subsamples of 10 seedlings out of 250 subsamples had tested positive. Further subsampling was not done. Seedlings were not raised to full-grown plants in order to check for viroid symptoms. Viroid sequence was identical to the sequence found in the tomato greenhouse in France (was considered additional evidence that the viroid was transmitted via seeds).
Elliott DR et al., 2001. Plant	49	New Zealand	PSTVd	10	S				P		Seed was suggested by the authors as source of inoculum. However, 1) first symptoms were observed near 'the access points of the glasshouse',

Disease 85, 1027.											2-3 months after planting. The finding near the 'access points' indicates introduction from plants outside of the glasshouse and NOT from seeds. 2) The authors report that PSTVd was identified in symptomatic plants by the Dutch Plant Protection Service. This is correct, however, the symptomatic plants had been analysed several months before and several months had passed since planting (Verhoeven, pers. comm.; Verhoeven et al., 2004), further reducing the chance that seeds were the source of inoculum. 3) Lebas et al. (2005) failed to transmit the isolated viroid by seed. 4) The sequence found in this tomato crop groups in the so-called cluster of Physalis peruviana-like PSTVd sequences (Verhoeven et al., 2004 and 2010b), mainly including isolates from Oceania. 5) PSTVd was reported in tomato crops in three more glasshouses during a survey held at 50 companies in New-Zealand: two in the vicinity of the first finding and one at a distance of 480 km without indicating that the plants in those glasshouses would have had the same seed source.
EPPO-Reporting Service 2004/006.	63	Germany	PSTVd	11		P				U	It was be assumed that an outbreak of PSTVd in a German tomato crop was introduced by imports of tomato planting material. However, no supporting evidence was provided. Moreover, it is unclear whether seeds were included as potential source of infection for the planting material.
EPPO-Reporting Service 2008/177.	58	Austria	PSTVd	12	S					U	The positive PCR-results from AGES in Austria could not be confirmed by tests at the NPPO of the Netherlands both in seeds and RNA from seeds sent by AGES. Testing seeds of the same lot by a German laboratory only gave negative results. NB testing in Austria was performed by nested PCR with self-developed primers.
EPPO-Reporting	74	UK	PSTVd	13			U			U	It was reported that the origin of the outbreak could not be identified.

Service 2011/202.											
EPPO-Reporting Service 2013/148.	75	The Netherlands	PSTVd	14			U			U	Back-tracing has not revealed the origin of the outbreak.
EPPO-Reporting Service 2013/236.	76	France	TASVd	15			U			U	It was only reported that TASVd was detected in asymptomatic plants of Brugmansia spp., Solanum jasminoides and S. lycopersicum (tomato). Details on origin are lacking.
Fagoaga C & Duran-Villa N, 1996. Plant Pathology 45, 45-53.	62	Spain	CEVd	16		P			P		Authors suggest that crops and other hosts may be sources of inoculum, especially when these plants are symptomlessly infected. The detection of CEVd in four different hosts (tomato, eggplant, carrot and turnip) in the same area suggests local sources of inoculum.
Fox et al., 2013. New Dis Rep 27, 8.	65		TCDVd	17		P			P		Quotation: A neighbour-joining tree showing the relationship of the outbreak TCDVd sequences to other TCDVd and PSTVd sequences suggests that the infections in the Norwegian samples may be related to samples obtained during the 2010 European-wide outbreak of TCDVd in petunias.
Hailstones et al., 2003. Aust Plant Pathology 32, 317-318.	59	Australia	PSTVd	18	S					U	No data have been provided that indicate that the viroid was introduced via seeds. Still the authors write that PSTVd can be seed borne and therefore suggest import regulations pertaining to tomato seed may need to be more stringent. However, only two plants of 100,000 were found infected by symptomatology. Since the sequence of AF536193 is quite similar to PSTVd sequences from Solanum jasminoides, this ornamental may have been source of inoculum too.
Ling KS & Bledsoe ME, 2009. Plant Disease 93, 839.	77	Canada	TPMVd	19			U			U	The authors report that the origin of viroid causing the outbreak in tomato is unknown.

Ling KS & Sfetcu D, 2010. Plant Disease 94, 1376.	67	USA, California	PSTVd	20			U		P		Authors do not known the source of infection and indicate both seeds and plants (ornamentals/potatoes). Personal information: based on own test results , PSTVd was known to occur in tomato plants in the respective glasshouse and surroundings, already several years before publication. The occurrence of the viroid in tomato plants both in the glasshouse and in its vicinity, indicates that these plants may have been the source of inoculum for the glashouse-grown tomato plants.
Ling KS & Zhang W, 2009. Plant Disease 93, 1216.	55	Mexico	TCDVd	21	S					U	The paper reports on both TCDVd and TPMVd in tomato. Diseased plants were first found in one, and later in two more greenhouses. It is not clear if both viroids were present in all greenhouses. The authors considered seed as source of infection for TCDVd as the viroid potentially can be seed transmitted in tomato. Moreover, they report that the close relationship between the Mexican TCDVd isolate from this report and TCDVd isolates from the USA isolates suggests that TCDVd in these two countries may share a common origin, likely seed. However, the source of infection in the USA is unclear (Ling et al., 2009). Moreover, since the first author tested samples from both locations in the same laboratory in the same period of time, cross-contamination in the laboratory should also be considered. If seeds were the sources of inoculum, the question remains why TPMVd – the viroid also found in Mexico - was not found in the USA. Considering the limited distribution of TPMVd, local plants in or near the glasshouse with the infected tomato crop may have been the source of infection.
Ling KS & Zhang W,	55	Mexico	TPMVd	22			U			U	The paper reports on both TCDVd and TPMVd in tomato. Diseased plants were first found in one,

2009. Plant Disease 93, 1216.											and later in two more greenhouses. It is not clear if both viroids were present in all greenhouses. The authors report that TPMVd had been identified in Mexico on papita (S. cardiophyllum) before, but they do not indicate a likely source of infection. Since Mexico probably is the area of origin of TPMVd, local plants in or near the glasshouse with the infected tomato crop may have been the source of infection. However, more evidence would be needed to state 'plants' as the orgin of infection.
Ling et al., 2013. Plant Dis 97, 148.	79	USA, North Carolina	PSTVd	23			U			U	Quotation: The broader geographic distribution of PSTVd on tomato in the USA, and the potential latent infection in potato and a number of ornamentals, emphasizes the need for better plant and seed health tests for viroids on these plants.
Ling KS et al., 2009. Plant Disease 93(10), 1075.	78	USA, Arizona	TCDVd	24			U			U	Quotation: The origin of TCDVd in this outbreak is not clear. The genotype identified first could have been introduced from a neighboring greenhouse where the disease was observed before 2006 and where this genotype also was identified in 2007. The second genotype may have been introduced from infected seed since TCDVd has recently been shown to be seed transmitted in tomato.
Ling KS et al., 2014. Plant Dis 98(5), 701.	80	Dominican Republic	PSTVd	25			U			U	Quotation: PSTVd from the Dominican Republic was likely introduced from a different source, although the exact source that resulted in the current disease outbreak remains unknown. It may be the result of an inadvertent introduction of contaminated tomato seed lots or simply from local wild plants.
Mackie et al., 2002. Austr Plant Pathology 31(3), 311-312.	10	Australia	PSTVd	26			U		P		Based on 219 nts of the viroid genome, it is most similar to the 'Naaldwijk' strain (X17268) (Puchta et al., 1991) and a strain of PSTVd from tomatoes in New Zealand (Elliott et al., 2001). So, the isolate is expected to group in the cluster with sequences showing highest identies with PSTVd sequences

											from Physalis peruviana, which indicates that local plants may have been source of inoculum. This hypothesis is further supported by the publication of Mackie et al., 2016.
Mackie et al., 2016. Eur J Plant Pathol 145, 433-446.	51	Australia	PSTVd	27		P			P		Slightly different sequences all belonging to the cluster of Physalis peruviana were found in various tomato and pepper crops, as well as in samples of nine species of weeds and wild plants such as Physalis angulata. According to the authors, the naturally occurring PSTVd inoculum reservoir in wild plants and weeds explains the occurrence of repeated PSTVd infections in different years in field crops of tomato, pepper and chilli.
Matsushita et al., 2008. J Gen Plant Pathol 74, 182-184.	11	Japan	TCDVd	28			U		U		The TCDVd genome (AB329668) from tomato is 100% identical to the genome of isolates from Petunia from Japan and some other countries. The fact that the same sequence was found in many Petunia selections originating from Japan, (Shiraishi et al., 2013; Verhoeven & Roenhorst, 2010) and that most TCDVd sequences (including sequences from tomato crops outside Japan) do not group in this petunia cluster, support the hypothesis that Japanese petunia plants most likely were the source of infection for tomato in Japan.
Matsushita et al., 2010. Eur J Plant Pathol 128, 165-170	56	Japan	PSTVd	29			U			U	In the publication no indications of the origin of infection are given. The sequence was identical to a sequence from Physalis florida (EU862231, Verhoeven et al., 2009. Plant Disease 93, 316–316).
Matsushita et al., 2010. Eur J Plant Pathol 128, 165-170.	56	Japan	TCDVd	30			U			U	In the publication no indications of the origin of infections are given. TCDVd sequence data are lacking.
Mishra MD et al., 1991. J	81	India	CEVd	31			U			U	In the publication no indications for the origin of infections are given.

Gen Virology 72, 1781-1785.											
Mumford RA et al., 2004. Plant Pathology 53, 242.	82	UK	PSTVd	32			U			U	The authors report that the origin of the infection is unknown and neither a likely source of infection could be deduced.
Navarro et al., 2009. Journal of Plant Pathology 91, 723-726.	13	Italy	PSTVd	33		P			P		Neighbouring ornamental plants of Solanum jasminoides were considered as the source of infection for tomato, because 1) PSTVd genomes in isolates from tomato and S. jasminoides were 100% identical, 2) the viroid sequence did not change after experimental transmission of PSTVd from S. jasminoides to tomato and 3) a phylogenetic analysis showed that the PSTVd sequence from tomato grouped with sequences from S. jasminoides.
Nixon T et al., 2009. New Disease Reports 19, 30.	60	UK	CLVd	34	S?					U	Infections found in tomato crops of cultivar Santa in four glasshouses. The same viroid sequence was found in plants from all greenhouses. This suggests that seeds (same variety / seed seed company) have been the origin of the infections. Moreover in France CLVd was identified in the same variety too (Steyer et al., 2010). Despite this circumstantial evidence for seed transmission, however, Fox and Monger failed to show seed transmission of CLVd raising 25,500 seedlings from the original seed lot!
Parrella et al., 2011. Acta Horticulturae 914, 149-152.	71	Italy	CLVd	35			U			U	In 2010 CLVd was identified in tomato cv Santa in a greenhouse where symptoms had been observed since 2008 (variety not reported). The sequence had 98% identity to the closest related sequence of CLVd reported at that time. It is unclear whether this outbreak is related to those in France (Steyer et al., 2009) and the UK (Nixon et al., 2009) because the variety showing symptoms in 2008 is not

											known. Based on the sequence identity of 98% a relation is not likely.
Parrella & Numitone, 2014. Plant Dis 98, 1164.	66	Italy	TASVd	36		P			P		Quotation: Sequences obtained were identical, showing the highest nucleotide identity (99.7%) with the TASVd isolate Sj1 (AM777161), identified in Germany on Solanum jasminoides. Quotation: The origin of this infection is still unclear, although based on the biological properties and sequence similarity, the To1-IT isolate probably originated from an ornamental species, most likely S. jasminoides, as recently reported for other tomato TASVd isolates, according to their biological and genetic features.
Puchta et al., 1990. Plant Molecular Biology 15, 509-511.	83	NL	PSTVd	37			U			U	PSTVd was identifeid in tomato and pepino plants from two glasshouses in the Netherlands. Viroid genome sequences were identical for the isolates from both tomato and pepino. The pepino plants had been imported from New Zealand and Greece (Crete) both as seeds and cuttings. NB The publication only reports seeds! PSTVd was assumed to be introduced via the pepino plants because the PSTVd infections in tomato were only observed in the two glasshouses where infected pepino plants were grown and because PSTVd was not known to occur in The Netherlands. [When later similar viroid sequences were reported in New Zealand pepino seeds/cuttings from New Zealand were considered as the source of infection (Verhoeven et al., 2004).]
Reanwarakorn et al., 2011. New Disease Reports 24, 6.	84	Thailand	PCFVd	38			U			U	No information on possible origin of the viroid.
Singh et al., 1999. J of Gen Virology 80, 2823-2828.	44	Canada	TCDVd	39			U			+	Quotation: The origin of TCDVd in greenhouse tomato crops has been hard to determine. The tomato seeds were produced in the Netherlands and were exported to the USA, from where the

											seeds were imported into Canada. Extensive efforts to demonstrate seed transmission or presence of viroid in the seed have failed. Subsequent batches of tomato seeds from the same sources have produced healthy crops. Therefore, seeds can be excluded as source of infection for this outbreak. NB. Later Singh (2008) showed seed transmission of TCDVd, probably with a sequence variant that was slightly different.
Steyer et al., 2010. Plant Pathol 59, 794.	85	France	CLVd	40			U			+	Maybe related to seeds (see Nixon et al., 2009), but seed transmission was not shown by Fox and Monger (2011). Moreover, the sequence of the isolate from France and those from the UK varied to some extent.
Van Brunschot et al., 2014. Eur J Plant Pathol 139, 1-7.	24	Australia	PSTVd	41	S			S			When PSTVd was identified in a crop of tomato cv Tiger, 370 remaining seeds from the original lot were raised for 10 weeks. One plant showed symptoms (shortened internodes and leaf purpling) and tested positive for PSTVd, whereas the viroid was not detected in the 369 symptomless plants. The viroid sequence in this tomato seedling was 100% identical to the sequence from the crop. Therefore, it was concluded that the viroid was introduced via the seeds.
Verhoeven et al., 2004. Eur J Plant Pathol 110, 823-831.	12	NL	CEVd	42		P			P		Seeds and tomato plants for planting are not likely sources of infection because all infections were found in different tomato varieties of different seed companies. Tracing back did not reveal other infections in the same seed lots or in other lots that were raised at nurseries who had raised lots in which later infections were found in glasshouses of tomato fruit producers. Therefore, seeds and tomato plants for planting were excluded as source of infection. The more recent findings of pospiviroids in symptomlessly infected

											ornamental plants indicate that these plants may have been sources of CEVd infection in tomato.
Verhoeven et al., 2004. Eur J Plant Pathol 110, 823-831.	12	Belgium	CLVd	43			U			U	No data available on origin
Verhoeven et al., 2004. Eur J Plant Pathol 110, 823-831.	12	NL	CLVd	44		P			P		Seeds and tomato plants for planting are not likely sources of infection because alle infections were found in different tomato varieties of different seed companies. Tracing back did not reveal other infections in the same seed lots or in other lots that were raised at nurseries who had raised lots in which later infections were found in glasshouses of tomato fruit producers. Therefore, seeds and tomato plants for planting were excluded as source of infection. The more recent findings of pospiviroids in symptomlessly infected ornamental plants indicate that these plants may have been sources of CLVd infection in tomato.
Verhoeven et al., 2004. Eur J Plant Pathol 110, 823-831.	12	NL	PSTVd	45		P			P		Seeds and tomato plants for planting are not likely sources of infection because alle infections were found in different tomato varieties of different seed companies. Tracing back did not reveal other infections in the same seed lots or in other lots that were raised at nurseries who had raised lots in which later infections were found in glasshouses of tomato fruit producers. Therefore, seeds and tomato plants for planting were excluded as source of infection. The more recent findings of pospiviroids in symptomlessly infected ornamental plants indicate that these plants may have been sources of PSTVd infection in tomato. Moreover, in 2010 phylogenetic analysis supported that PSTVd isolate 21008470 had originated from Solanum jasminoides (Verhoeven et al., 2010b). Two PSTVd outbreaks in The Netherlands from this publication are not

												included, as they were already included in this table via the publication of Puchta et al. (1990). Similarly, a PSTVd outbreak from New Zealand was not included as that was already included via the publication of Elliott DR et al., 2001. Plant Disease 85, 1027.
Verhoeven et al., 2004. Eur J Plant Pathol 110, 823-831.	12	USA	TCDVd	46			U			U	No data available on origin.
Verhoeven et al., 2006. Plant Disease 90, 528.	86	Tunisia	TASVd	47			U			U	No data available on origin.
Verhoeven et al., 2007. Plant Disease 91, 1055.	87	Belgium	PSTVd	48			U			U	First symptoms were observed ca. 6 month after the tomato plants had been received from a Dutch nursery. No PSTVd was found in glasshouses of other growers who had purchased tomato plants from the same nursery.
Verhoeven et al., 2012. Eur J Plant Pathol 133, 803-810.	64	NL	TASVd	49		P			P		The occurrence of the same TASVd genotype in various ornamental crops and a single crop of tomato, suggests a common origin, similarly as reported for PSTVd (Navarro et al., 2009; Verhoeven et al., 2010). This hypothesis was further supported by successful mechanical inoculation of the same sequence variant of TASVd from Lisianthes rantonnetii and Solanum jasminoides to tomato.
Walter et al., 1980. Ann Phytopathol 12, 259-75.	88	Ivory Coast	TASVd	50			U			U	Poor identification and no data available on origin.
					11	10	29	2	14	34	

[1] Source of infection assumed by the authors. 'Seed' covers the seeds from an infested lot. 'Plants' covers both infected plants for planting of the infected crop and infected wild or cultivated plants, not belonging to the plants of the infected crop. 'Unclear' is used when no source of infection is reported or in case of contradictory data.
[2] Deduced source of infection based on new interpretation of past and current conceptions. Further details are given in the column 'Comments'.
